# “Worm within worm”: acute appendicitis containing an adult Ascaris lumbricoïdes

**DOI:** 10.1259/bjrcr.20210035

**Published:** 2022-01-13

**Authors:** Ibrahima Niang, Coumba Khadija Dieng, Papa Malick Dibor Diouf, Cheikh Tidiane Diop, Ibrahima Bocar Welle, Abdourahmane Ndong, Serigne Ahma Mbacké Dia, Papa Balla Sarr, Ousmane Ndiaye, Richard Bazogo Sinake, Aissata Ly Ba

**Affiliations:** 1 Department of Radiology, Fann University Hospital Center, Dakar, Senegal; 2 Department of Radiology, Albert Royer Hospital, Dakar, Senegal; 3 Department of Surgery, Albert Royer Hospital, Dakar, Senegal; 4 Department of Surgery, Gaston Berger University Saint-Louis, Saint-Louis, Senegal

## Abstract

Appendicitis is the first etiology for abdominal surgical emergency. Ascariasis is the most common helminth infection in tropical countries where it is endemic. The ability of intestinal helminths to wander through the digestive system means that it can end up in the appendix lumen and lead to appendicitis by luminal obstruction. However, this presentation is still rarely described in the literature. In fact, most of the diagnoses of roundworm associated with appendicitis are made retrospectively by the discovery of its eggs on the pathological examination of an appendectomy specimen. We report the case of a 7-year-old patient living in a tropical area who consulted for acute right lower quadrant pain. The ultrasound suspected the diagnosis of appendicitis and also revealed multiple intestinal worms including one in contact with the inflamed appendix. Surgical exploration confirmed appendicitis associated with roundworm partly in the appendicular lumen through a perforation.

## Introduction

Appendicitis is the first etiology for abdominal surgical emergency, with a lifetime incidence of 7–14%.^1^ Acute appendicitis is often related to an obstruction of its lumen with multifactorial causes including fecaliths, lymphoid hyperplasia, tumors and intestinal parasites.^2,3^ Ascariasis is an infection caused by a giant roundworm called *Ascaris lumbricoides*, with an estimated 760 million cases worldwide.^4^ It is particularly endemic in tropical and underdeveloped countries and mainly infects malnourished children living in unsanitary conditions.^5^ The surgical manifestations of abdominal roundworm are multiple and mainly related to the capacity of the worm to wander through the digestive and biliary tract.^6,7^ Cases of appendicitis associated with roundworm are infrequent. Besides, the responsibility of roundworm for the occurrence of acute appendicitis remains debated.^8,9^ We report the case of a 7-year-old patient living in a tropical area who consulted for acute right lower quadrant pain. The ultrasound suspected the diagnosis of appendicitis and also revealed multiple intestinal worms including one in contact with the inflamed appendix. Surgical exploration confirmed appendicitis associated with roundworm partly in the appendicular lumen through a perforation.

This form of presentation of appendicitis associated with roundworms is exceptional in the literature.^10,11^

## Case description

**Figure 1.**
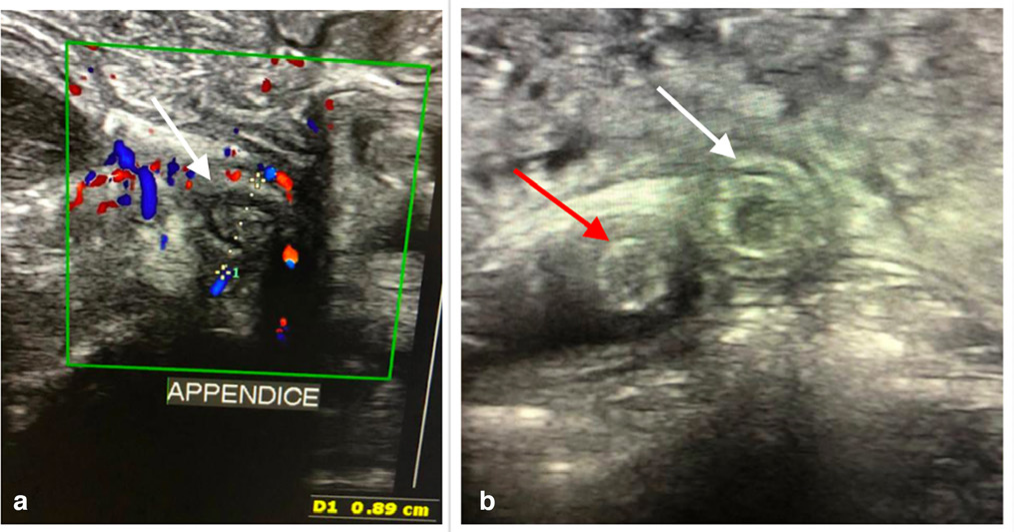
Axial view of the right iliac fossa with the high-frequency probe (A)appendix increased in diameter measuring 8.9mm (white arrow) with a dedifferentiated wall and hyper vascularized on color Doppler (B) double target-sign corresponding to the appendix (white arrow) and the Ascaris in contact (red arrow).

**Figure 2.**
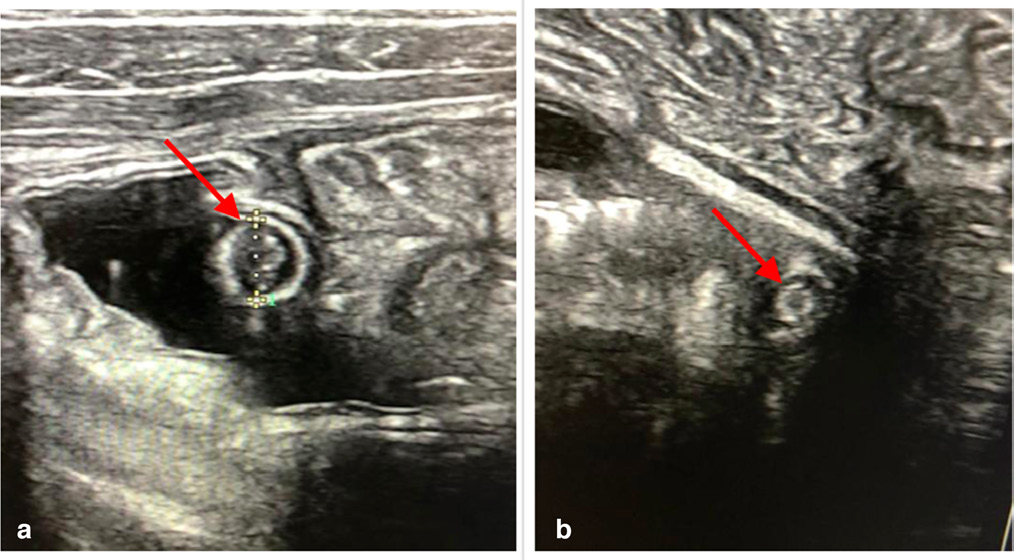
Axial view of the intestinal loops with the high-frequency probe. (A and B) Axial section of Ascaris in the intestinal loops giving a target-sign (red arrows) (A) intraoperative view after McBurney incision showing an intestinal loop (white star), inflamed appendix (white arrow) and roundworm (red arrow) partly in the appendix through a perforation (B) post-operative image showing the appendectomy specimen (white arrow) and the Ascaris measuring 20 cm long (red arrow).

A 7-year-old male patient with no medical history was received at the emergency department for intense right lower quadrant pain evolving for 2 days and associated with nausea and vomiting. His general condition was preserved without any sign of dehydration. The temperature was elevated to 37.8°C and the rest of the vital signs were within normal ranges. Physical examination revealed tenderness of the right lower quadrant without abdominal distension or palpable mass. The rest of the examination was normal. The biological tests showed leukocytosis at 11700/mm^3^ (neutrophil at 80%) and a high level of CRP at 20 mg/L. With the suspicion of acute appendicitis, an abdominal ultrasound was required. It revealed an appendix located at the right lower quadrant. It was dedifferentiated, thickened with a diameter of 8.9 mm associated with hyper echogenicity of the surrounding fat and hypervascularization on Doppler (Figure 1A). In contact with the appendix, another cylindrical structure was visualized giving a target appearance on axial sections (Figure 1B). Several other similar structures were also found, mobile within the intestinal loops (Figure 2A–B) without any sign of obstruction. There was no other anomaly identified. Totally, the ultrasound concluded with acute uncomplicated appendicitis of the right iliac fossa associated with several intestinal worms, one of which was in contact with the appendix.

**Figure 3.**
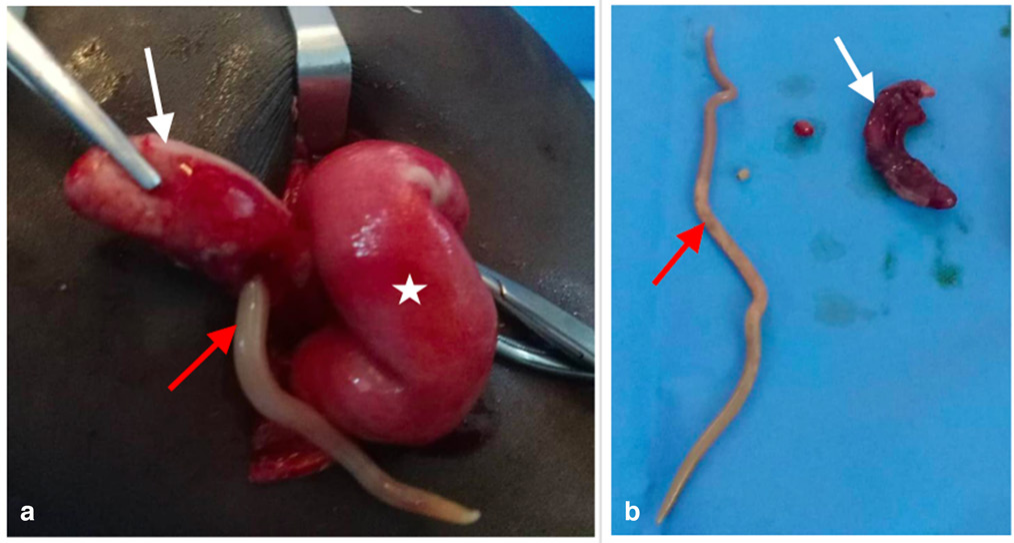
Images in the operating room.

The patient was taken to the operating room for an open appendectomy (McBurney incision). Surgical exploration showed an appendix with edema and inflammation with roundworm halfway into the lumen of the appendix through a perforation (Figure 3A). The appendectomy was completed and a 20 cm living roundworm was removed (Figure 3B). The patient was discharged after 3 days with anthelminthic treatment and the post-operative course was uneventful.

## Discussion

The earliest anatomical descriptions of the appendix date back to the 16th century. In 1561, Fallopian (which gave its name to the uterine tubes) compared the appendix to a worm, hence the name *appendix vermiformis*.^12^ Appendicitis is the most common condition leading to an abdominal surgical emergency.^1^ Its pathophysiology involves luminal obstruction which leads to an increase in intraluminal pressure leading to mucosal ischemia. With vascular congestion, the appendicular mucosa becomes hypoxic and begins to ulcerate, resulting in compromise of the mucous barrier and leading to invasion of the appendicular wall by intraluminal bacteria. Additionally, stasis of intraluminal contents leads to bacterial overgrowth in the inspissated mucus.^2,8^

Ascariasis is endemic in underdeveloped areas and particularly affects children.^13^ Its clinical manifestations are polymorphic depending on the phase of infection. During the gastrointestinal phase, digestive signs are more common and may mimic acute abdominal emergency.^14^ However, they can be responsible for emergency abdominal surgery due to digestive or biliary obstruction.^6,7^ In fact, the adult worms roam the digestive system and this is how roundworms can end up in the appendicular lumen. It can cause appendicitis by obstruction according to the pathophysiological mechanism already described. However, the direct responsibility of roundworm in the occurrence of appendicitis remains debated.^8,15^ Suspected appendicitis caused by roundworm is most often diagnosed by finding roundworm eggs on appendectomy specimen.^16^ Hence, the importance of anatomopathological examination of all appendectomy specimen, particularly in tropical countries where helminth infections are endemic. In our patient, the ultrasound helped to identify a roundworm in contact with the appendix as well as other roundworms in the lumen of the digestive loops. These ultrasound signs in favor of roundworm have been described since the 1980s^17^ and should be known by radiologists and ultrasonographers, particularly in tropical areas. These ultrasound aspects are described as: thick echogenic strip with central anechoic tube; multiple linear or curvilinear echogenic strips without acoustic shadowing; “parallel lines”; “railway track” sign; “3-line”or “4-line” sign on longitudinal scan and a “doughnut” or “target” sign and “bull’s eye” appearances on transverse scan.^14,17^ It is also important to note that on ultrasound, especially on axial slices, the appendix is somewhat similar to *Ascaris lumbricoides*, both giving a target appearance.^18^ So, it is important to know the difference by highlighting the attachment of the appendix to its base in the cecum. The other differential diagnoses on ultrasound are nasogastric or jejunal feeding tube, surgical drainage tube, a ventriculoperitoneal shunt, or even anormal small bowel during peristalsis.^17,19^ Surgical exploration revealed an inflamed appendix containing roundworm through a perforation. This suggests that the roundworm entered the appendicular lumen and left it by puncturing it after causing appendicitis. After the appendectomy, our patient underwent anthelmintic treatment, especially as other intestinal Ascaris were identified on ultrasound. This treatment is still necessary for any appendicitis associated with roundworm in order to eliminates all potential other infraclinical locations.

In conclusion, the possibility of appendicitis caused by Ascaris in its adult form needs to be considered particularly in patients from endemic areas. In addition, radiologists and ultrasonographers should be familiar with the ultrasound aspects of roundworm and also know how to differentiate it from the appendix itself.

## Learning points

1. Ascariasis can mimic acute appendicitis, and is therefore one of its differential diagnoses.
2. However, as in this case, appendicitis can exceptionally be caused by roundworm.
3. In a tropical area with endemic roundworm, this diagnosis should be considered and looked for with ultrasound
